# Correlation between Antioxidant and Anti-Osteoporotic Activities of Shilajit Loaded into Chitosan Nanoparticles and Their Effects on Osteoporosis in Rats

**DOI:** 10.3390/polym14193972

**Published:** 2022-09-23

**Authors:** Fawzia A. Alshubaily, Ebtihaj J. Jambi

**Affiliations:** Biochemistry Department, Faculty of Science, King Abdulaziz University, Jeddah 21551, Saudi Arabia

**Keywords:** nanopolymers carriers, nano-conjugation, shilajit extract, in vivo, antioxidant biomarkers

## Abstract

Various therapies for osteoporosis successfully reduce bone loss and fractures, but they mostly do not contribute to new bone structures and adversely affect patients. Shilajit is a natural mineral substance comprised of multi-components, with proved efficacy to improve immunity, antioxidant activity, and disease resistance. In the present study, various effects of shilajit water extract (SWE) on bone development and its management were determined in experimental glucocorticoid-induced osteoporotic rats. The fabrication of nanochitosan (NCT) and NCT conjugation with SWE were conducted and evaluated as enhanced formulations for treating osteoporosis. NCT and SWE/NCT had mean particle diameters of 196.4 and 248.4 nm, respectively, with high positivity charging and stability. The biochemical and anti-osteoporotic effects of SWE and SWE/NCT conjugates were investigated on different groups of compromised rats. Five groups each including six adult albino female rats were formed and treated for a duration of eight weeks with SWE and SWE/NCT conjugate. Significantly improved serum calcium, phosphorus, osteocalcin, and calcitonin levels but decreased hydrogen peroxide, IL-6, and antioxidant biomarkers were recorded in all SWE- and SWE/NCT-treated groups; the SWE/NCT treatment was most effectual treatment. These results suggest that SWE and SWE/NCT may cause anti-osteoporotic activity by reducing oxidative stress, IL-6, and H_2_O_2_ while restoring antioxidant levels. The conjugation of SWE onto NCT is highly recommended for augmenting their activities.

## 1. Introduction

Shilajit is a mineral pitch, which is a sticky, black, and tar-like substance in nature. It is formed by the natural process by breaking down minerals and plant matters and used traditionally for a long time in India and Tibet, now reported to be used in many other countries [1]. Shilajit strengthens bones, improves the transfer of phosphate, magnesium, and calcium in the bones, and prevents the occurrence of osteoporosis [2]. Many recent studies have validated shilajit as a panacea for oriental medicine and validated its various properties. Shilajit is an ancient traditional medicine possessing several pharmaceutical properties, which cured many pathological conditions in addition to showing anti-osteoporotic effects [3].

Shilajit is characterized as a multi-component natural mineral medicine, traditionally used for the treatments of libido, injury, aging, immunity, obesity, blood sugar, and arthritis [4,5]. Shilajit is a natural blackish-brown herbal compound, which contains fulvic and fulvic acids and is considered safe to be taken orally as part of a person’s everyday diet [2]. It may be used rationally in future clinical studies investigating its effectiveness to cure various human ailments.

The World Health Organization (WHO) characterizes osteoporosis as having a bone mineral density at or below 2.5 ± SD. The diagnostic criteria of the WHO also include qualitative bone estimating, bone tissue deterioration, and disrupted bone structures [6]. There are more than nine million fractures that happen annually in the human population, approximately one every 3 s due to osteoporosis [7]. Osteoporosis is a major public health concern affecting medical, social, and economic aspects of life; females can habitually suffer fractures six times more than males (6:1 ratio) [8]. The “International Osteoporosis Foundation” reported a worldwide population of more than 200 million females above the age of 60 years who suffer from osteoporosis. For postmenopausal osteoporosis, a dose-dependent treatment with Alendronate and Risedronate (Bisphosphonates) is considered as the first-line therapy, since they increase the bone mineral density in the spinal cord and hip regions [6].

Currently, many other medicines are available to treat osteoporosis, but the choice of a specified therapy must follow the recommended guidelines. The reason for this caution is that there are numerous classical medicines available in markets which claim to be therapeutic and efficacious against osteoporosis but cause some serious health concerns as well. Hence, studies are required to discover other traditional medicines, which are safe and therapeutic in nature offering more panaceas without causing any health concerns to the human population. The use of shilajit could be influenced by its bioavailability and potential toxicity in high-dose treatments [9].

Chitosan (the bioactive deacetylated form of chitin) was recurrently and successfully employed in many fields that related to human health, nutrition, pharmaceuticals and environment [10]. The biodegradability, bioactivity and functionality of chitosan promoted its applications in more fields that involved its combinations with further bioactive molecules, e.g., antibiotics, plant extracts, nanomaterials, growth promoters and nutraceuticals [11]. The transformation of chitosan into its nanoforms provides more functionalities and capabilities to carry/conjugate further molecules, which were recurrently verified using various phytoconstituents, nanomaterials, antibiotics, hormones, enzymes, vitamins and proteins [12,13].

Hence, the following study was designed to determine therapeutic applications of shilajit, its effectiveness to rejuvenate bone mineral mass, bone structures and various other bone parameters in the experimental rats under laboratory conditions. The construction of nanocomposites from nanochitosan and shilajit, their characterization and evaluation were additionally investigated.

## 2. Materials and Methods

### 2.1. Experimental Materials and Animals

#### 2.1.1. Chemicals

Shilajit was purchased from Natural Spirit Trading, Riyadh, Kingdom of Saudi Arabia. Chitosan (CAS Number 9012-76-4; medium molecular weight) was supplied from Sigma-Aldrich (St. Louis, MI, USA) with a deacetylation degree of 75–85% and a viscosity of 200–800 cP in 1% acetic acid solution. Tri-polyphosphate (TPP) and acetic acid were provided from Sigma-Aldrich (St. Louis, MI, USA).

#### 2.1.2. Animals

All the regulations laid down by the Laboratory Animal Resources, Institute of National Research Council, and Commission on Life Sciences were compiled in experimenting with the live animals [14]. Female adult albino rats of the Sprague Dawley strain (n = 24), weighing approximately 200 ± 5 g, were purchased from the King Fahad Research Center, King Abdulaziz University, Jeddah. They were housed individually in wire cages under healthy conditions at a room temperature of 23 ± 2 °C. The animals were fed a basal diet with water ad libitum in accordance with the nutrient supplies of Laboratory Animals [14], and kept under surveillance for five days before conducting any experiments with them.

#### 2.1.3. Basal Diet

The basal diets for the experimental animals, including mineral and vitamin mixtures, were prepared based in combination of various nutrients as suggested [14]. It was comprised of 10% each of protein and corn oil, 1% and 4% of vitamin and mineral mixtures, respectively, 0.2% of choline chloride, 0.3% of methionine, 5% of cellulose, and 69.5% of corn starch.

### 2.2. Nanochitosan (NCT) Preparation and Conjugation

#### 2.2.1. Nanoconjugates Synthesis

NCT preparation was conducted according to a formerly described protocol [15], which involved dissolution of chitosan at a percentage of 0.1% (*w*/*v*) in 1% aqueous acetic solution, then an equal volume of TPP aqueous solution (0.05%, *w*/*v*) was dropped slowly (18 mL/h) into chitosan solution. The mixture of solutions was harshly stirred (at 780× *g*) throughout the addition of TPP dropping and for 90 min after. The formed NCT were collected through centrifugation (5500× *g* for 30 min) and the pellet was washed with distilled water, re-centrifuged and freeze-dried.

For shilajit extract (SWE) conjugation with NCT, shilajit was dissolved in deionized water (0.1%, *w*/*v*) and an equal volume was added to chitosan solution before TPP solution dropping, stirred for 125 min (at 650× *g*) and then the rest of steps were conducted, as illustrated above.

#### 2.2.2. Nanomaterials Characterization

The inter-molecular bonding and biochemical groups in examined molecules/conjugates (e.g., SWE, NCT and SWE/NCT) were characterized using infra-red analysis (Fourier Transform Infrared “FTIR” Spectrophotometer, Perkin Elmer 200, Waltham, MA), after mixing the molecules with KBr, and their spectra were detected within a wavelength range of 4000–450 cm^−1^ at 25 °C. The surfaces ultrastructure of nanomolecules, e.g., NCT and SWE/NCT, were elucidated with SEM imaging “Scanning Electron Microscopy; JEOL JSM- IT510, Tokyo, Japan”, after coating samples with gold/palladium. The size/charge of nanocomposite particles was further judged through the DLS technique “Dynamic Light Scattering, Malvern Zetasizer, Malvern Instruments, Malvern, UK”, after dispersing NPs in deionized water and their sonication.

The used biological samples, e.g., prawn and macroalgae (*Sargassum linifolium*), were amassed from the western coast of Saudi Arabia at the Red Sea, within 35°65′ E–37°12′ N and 26°03′ N–31°15′ N. All samples were identified morphologically by specialized marine biologists in the “University of Tabuk, Saudi Arabia”.

### 2.3. Experimental Design

Five groups of experimental animals were formed based on random selection and each group included six rats, which were kept on the basal diet until they were used for experiments. The first group of rats termed as Group 1 was designated as a negative (-ve) control group, which received a single dose of 0.9% saline daily for eight weeks. The other groups received an intramuscular subcutaneous injection of methylprednisolone (MP; 30 mg·kg^−1^ in a 0.9% NaCl solution) on daily basis for eight weeks in order to produce glucocorticoid-induced osteoporosis (GIO) [16]. These groups were designated as the members of the untreated positive (+ve) control group (Group 2). After inducing osteoporosis, experimental rats were administered daily by gavage with 1 mL of 0.9% diluted saline solution, and food and water were provided ad libitum. Each rat was examined daily for the development of any symptoms and general health conditions. Each rat was housed in a separate iron cage, kept at 23 ± 2 °C, relative air humidity of 60–70%, and 12 h:12 h light-dark cycles during the experimentation. Treatment groups were classified as follows: Group 3 (low SWE dose), where GIO-afflicted rats were treated with SWE at 150 mg/kg for 8 weeks and Group 4 (high SWE dose), where GIO-afflicted rats were treated with SWE at 250 mg/kg in a distilled water vehicle for 8 weeks. In fifth group (Group 5, SWE/NCT), the GIO-afflicted rats were treated with SWE/NCT at 500 mg/kg dose for 8 weeks. The experiments in the current study were designed and conducted according to the “Helsinki Declaration” guidelines, revised in 1975 [14].

### 2.4. Blood Sampling and Biochemical Analysis

#### 2.4.1. Sample Collection

After the experiments were terminated, the rats were anesthetized with diethyl ether and sacrificed to obtain their livers and bones. Blood samples were collected into tubes with or without an anticoagulant (sodium citrate) depending on the method. Centrifuged at 2000× *g* for 10 min at 4 °C, the blood samples were stored frozen at −20 °C for subsequent analytical analysis.

#### 2.4.2. Bone Sample Preparation

Adhering to the muscles, the left tibias were detached, dissected out of the animal, cleaned, and stored at −80 °C. When the samples were ready, total antioxidant capacity (TAC), superoxide dismutase (SOD), and malondialdehyde (MDA) were estimated.

#### 2.4.3. Lipid Peroxidation Estimation

To estimate lipid peroxidation, Ski Lifts Assay kits were obtained from Cayman Chemical Company, USA. They were used to estimate MDA levels in the bones. After dissection, the left tibia was homogenized by grounding and mincing bone samples in a porcelain mortar. A 1.5 mL centrifuge tube was filled with 25 mg of minced bone tissue before blending it with 250 mL of a buffer solution with protease inhibitor (EDTA) for Radioimmunoprecipitation Assay (RIPA). The minced bone sample was further homogenized with a 40 V sonicator for 15 m at 4 °C before centrifuging at 1600× *g* for 10 min. The MDA levels were estimated spectrophotometrically at 540 nm from the supernatant collected from centrifuged sample stored at −80 °C [17].

#### 2.4.4. SOD and TAC Measurement

Phosphate-buffered saline with pH 7.4 was used to perfuse homogenized left tibias and to remove any platelets or clumps from the tissues. A small sample of 0.25 g bone was squashed with a mortar and pestle on the ice. The macerated bone tissues were then placed in a 10 mL tube filled with 2 mL of 20 mM HEPES buffer. The HEPES buffer was comprised of 210 mM Mannitol, 1 mM EDTA, and 70 mM sucrose and the pH was adjusted at 7.2. The tissue was placed on the ice and the tissue sample was homogenized again in a homogenizer for better blending. The blended sample was then centrifuged at 1500× *g* at 4 °C for 5 min and the resulting supernatant was stored in a tube for subsequent examinations. Superoxide dismutase activity (SOD) was determined by the methods of Dechatelet et al. [18]. The total antioxidant capacity was measured by using a commercial kit obtained from Bio-diagnostic, Giza, Egypt.

#### 2.4.5. Biochemical Parameter Determination

Calcitonin (CT), osteocalcin (OC), and IL-6 levels were analyzed by implying a sandwich solid-phase enzyme-linked immunosorbent assay. The commercially available kits for CT (Cusabio), OC (Elabscience), and IL-6 (R&D Systems) were used to run immunosorbent assays. Levels of Hydrogen peroxide (H_2_O_2_) were assessed with the described methods [19].

#### 2.4.6. Histopathological Study

Liver and kidney specimens were collected and immediately fixed with 10% buffered formalin. They were dehydrated in 70, 80, and 90% alcohol concentrations, cleared in xylene and embedded in paraffin wax for sectioning. The sections of 4–5 μm in size were cut and stained with hematoxylin and eosin dyes.

### 2.5. Statistical Methods

SPSS 16.0 (SPSS, Inc., Chicago, IL, USA) was used to analyze the data. Student’s *t*-test was used to determine statistical differences between the means at a 95% confidence limit (*p* < 0.05). Wherever it is necessary, the data are presented as an arithmetic mean with standard deviations.

## 3. Results 

### 3.1. Nanoconjugates Formation and Characterization

The SWE and NCT were successfully generated and conjugated in our study; the biochemical and structural analysis of generated molecules validated their formation and interaction.

**Figure 1:**
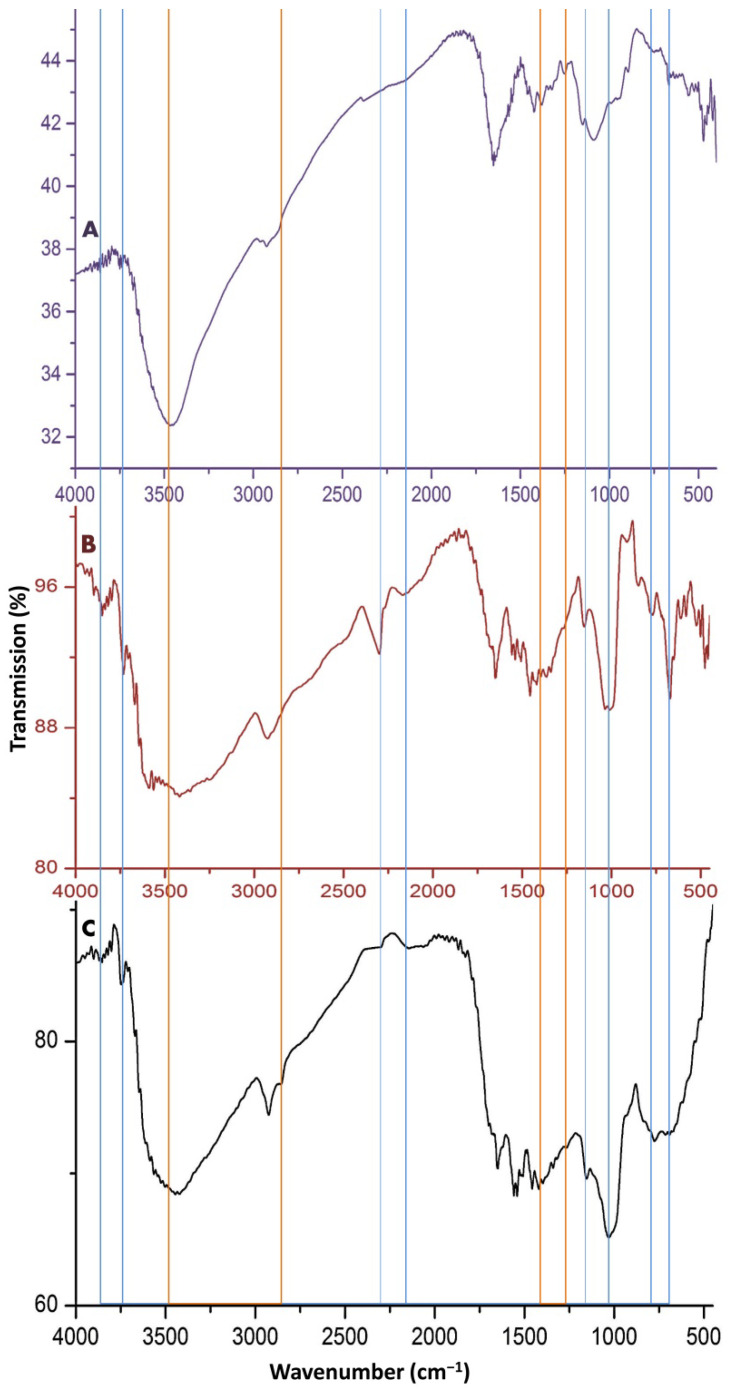
The infrared analysis (FTIR) of employed molecules in the current study, including (**A**) nanochitosan, (**B**) shilajit water extract and (**C**) their composites. The blue vertical lines indicate the derived bonds from SWE and the red lines indicate the derived bonds from NCT in the SWE/NCT spectrum.

The infrared (FTIR) spectroscopical analyses of invented molecules are presented in Figure 1. In the IR-spectrum of NCT (Figure 1A), the band at approximately 3422 cm^−1^ corresponds to the stretched N–H and O–H molecular hydrogen bonds. The detected NCT biochemical bonding correspond to the following bands: ~1653 cm^−1^ (C=O stretching of amide I); 1323 cm^−1^ (C–N vibrated stretching); 1144 cm^−1^ (C–O–C bridge asymmetric stretching); 1022 cm^−1^ (stretched C–O). The detected peaks at 1144 and 1022 cm^−1^ correspond to the overlapping of C–O and NCT formation after interaction between NH4 groups in NCT and PO4; the peak at 1193 cm^−1^ corresponds to the stretched P=O bond. 

Regarding the SWE spectrum (Figure 1B), the wide band at approximately 3400 cm^−1^ is indicative of the stretched hydrogen bond O–H, whereas the peak at 2932 cm^−1^ corresponds to the aliphatic C–H stretch. The band in the region of 1646 cm^−1^ correspond to the aromatic C=C bond, C=O bond H– of conjugated ketones, while the bands at 1538 and 1419 cm^−1^ indicated O–H bending/vibrations of alcohols/carboxylic acids. At 1089 cm^−1^, the band corresponds to the stretched C–O, which indicates the occurrence of polysaccharide-like molecules. Relevant studies concerning the FTIR spectra of NCT and SWE are presented in the “Discussion” section, to support the obtained findings here.

The spectrum of the composite (Figure 1C) emphasizes the occurrence of numerous chemical bonds that were derived from NCT and SWE in the SWE/NCT spectrum (indicated by the vertical lines in the figure).

**Figure 2:**
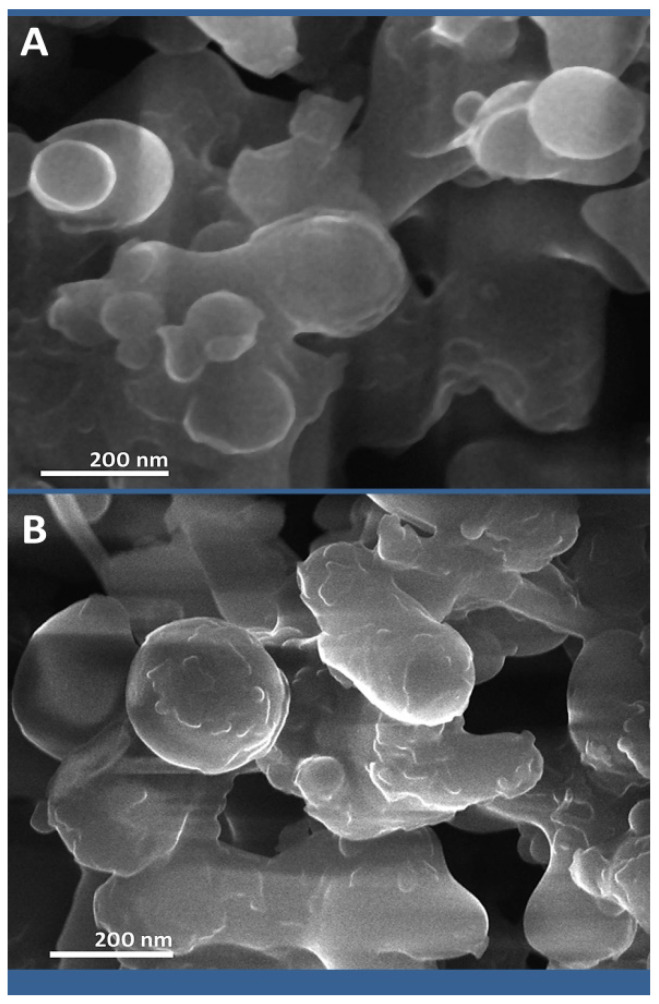
Scanning microscopy imaging of (**A**): synthesized nanochitosan and (**B**): its conjugates with shilajit water extract.

The structural features generated nanoparticles (NCT and SWE/NCT) that are emphasized in Figure 2. The plain and loaded NCT particles with SWE had inconsistent shapes (mostly semi-spherical and spherical) and their estimated particle size ranged from 48.6 to 377.4 nm for NCT and from 65.4 to 451.5 nm for SWE/NCT composites, which are within the nano scale of nanopolymers (1–1000 nm).

The DLS analysis of nanoparticles indicated that for NCT, the computed particles size range was 42.4–368.5 nm, with a mean particle diameter of 196.4 nm, whereas the diameter range of SWE/NCT particles was 63.7–446.8 nm, with a mean particle diameter of 248.4 nm. The particle surface charge for the nanopolymer molecules was +38.4 and +35.3 mV for NCT and SWE/NCT, respectively.

### 3.2. Serum Analysis

Table 1 shows serum concentrations of calcium, phosphorus, OC, CT, and IL-6 for all the groups. Serum levels of calcium, phosphorus, and CT were decreased in Group 2 when compared that with Group 1.

The increase in their levels was found the highest after high-dose treatment (Group 4) as compared to low-dose treatment (Group 3). However, the serum concentration of OC and IL-6 was higher in Group 2 as compared to Group 1 (*p* < 0.05). Regarding different shilajit doses affecting the osteoporosis model, the lowest levels of both OC and IL-6 were in Group 4. Group 3 showed relative improvement in parameters due to relative increases in calcium, phosphorus, and CT and decreases in OC and IL-6 as compared to Group 4. The best scores were reported for Group 5 (treated with SWE/NCT composites), where it had the highest levels of Ca, P, CT and the lowest levels of OC and IL-6 among the GIO groups.

### 3.3. Oxidative Stress Indices and Bone Tissue Antioxidants

Changes in oxidative stress markers for all groups are presented in Table 2, where Group 2 demonstrates significantly decreased levels of TAC and SOD while showing increased MDA and H_2_O_2_ levels in Group 1.

The TAC and SOD levels increased for both treatment groups but MDA and H_2_O_2_ levels decreased as compared to Group 2. A significant decrease was found in the measured TAC and SOD levels in the serum by glucocorticoid-induced osteoporosis (GIO) (Group 2). However, the concentration of measured antioxidant biomarkers in Group 4 was significantly higher (*p* < 0.05) than they were measured in Group 2 animals. The levels of MDA and H_2_O_2_ were lower in Group 4 than in Group 3 (*p* < 0.05). The group treated with SWE/NCT composite was superior in oxidative stress markers levels, which had the significantly highest TAC and SOD levels and lowest MDA and H_2_O_2_ levels among the GIO groups.

It was found that calcium, phosphorus, TAC, serum calcium, serum phosphorus, and SOD were positively correlated with each other. Highly positive correlations were found between CT, SOD, and TAC (*p* < 0.05). Contrastingly, negative correlations were found between IL-6, serum calcium, phosphorus, CT, TAC, and SOD, and between SOD MDA, and H_2_O_2_ (*p* < 0.05) (Table 3).

### 3.4. Histopathological Investigations

**Figure 3:**
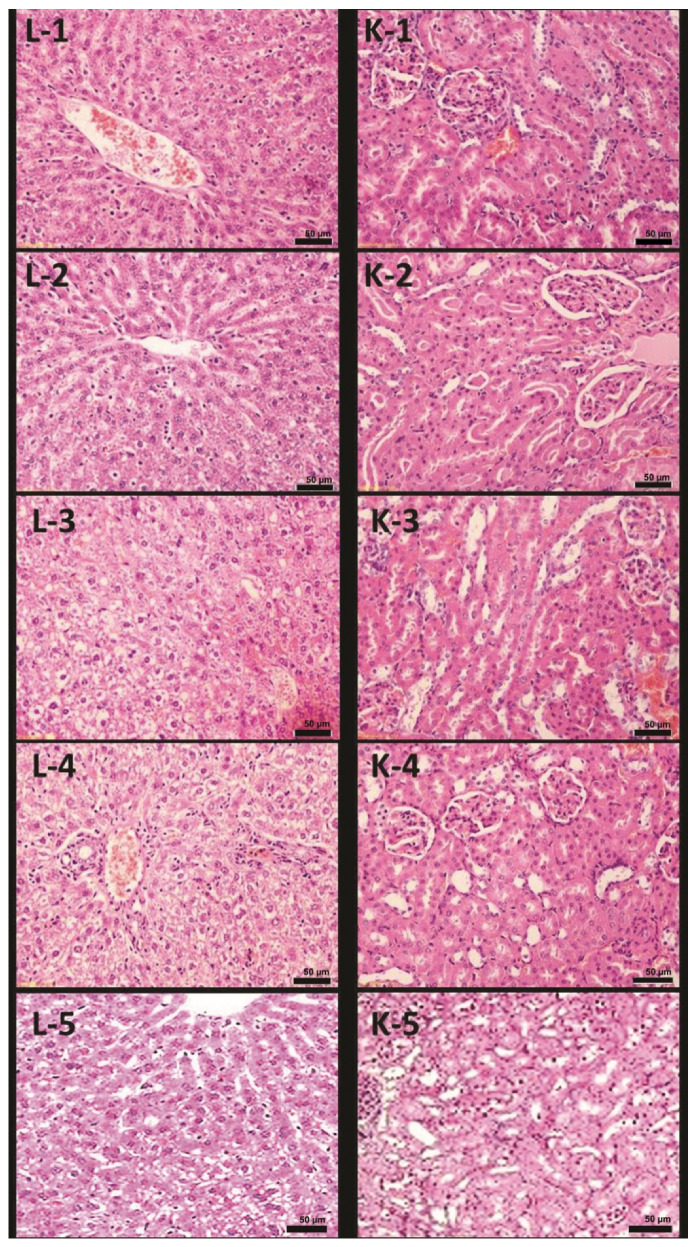
H and E-stained microscopic images of rats’ hepatic (**L**) and renal (**K**) tissues in the groups under study. **1**: the negative control group (group 1); **2**: the positive control group (group 2); **3**: the low-dose-SWE-treated group (group 3); **4**: the high-dose SWE-treated group (group 4); and **5**: the SWE/NCT-treated group (group 5).

Microscopic examination of the liver from Group 2 revealed hepatocytes, central vein, and sinusoids, demonstrating morphological changes that differed from those seen in Group 1. Distinct differences included ballooning degeneration of hepatocytes, moderate inflammation, central vein, and sinusoidal congestion (Figure 3(L2)) as compared to the normal hepatic tissue as shown in Figure 3(L1). Examination of the kidneys found glomerular tuft shrinkage and moderate tubular atrophy in Group 2 (Figure 3(K2)) when compared with the normal renal tissues of Group 1 (Figure 3(K1)).

Microscopic examination of the liver from Group 3 revealed inhibited inflammatory reactions despite recognizable mild inflammation. The histological features of hepatocytes and blood sinusoids were similar to those of Group 1 (Figure 3(L3)). Significant improvement of the renal tissue was also noted in Group 3 as compared with that found in Group 2. There were no clear significant pathological changes observed except that histological structure appeared similar to Group 1 (Figure 3(K3)).

Microscopic examination of the liver collected from Group 4 revealed inhibited inflammatory reactions and significant improvement in histological changes in the renal tissues as compared to that of Groups 2 and 3. Hepatocytes and blood sinusoids were most closely resembled the control group (Figure 3(L4)). There were no apparent significant pathological changes, and histological structures appeared closest to DEXA scans of Group 1 (Figure 3(K4)).

Small differences in the animal body composition due to three types of dietary treatments as shown by full-body scans by DEXA are presented in Table 3. Sucrose and honey-fed rats showed bigger lumbar spine areas as compared to the rats fed with a sugar-free diet (*p* < 0.05). As compared to the rats administered with a sugar-free diet, the honey-fed rats showed increased bone mineral density (BMD) (*p* < 0.05). Similarly, the rats when fed with sucrose for twelve months, they showed a higher percentage of total body fat (34.7%) in comparison to honey-fed animals (*p* < 0.05). No significant differences were found in the body fat levels when the animals were given honey-rich or sugar-free diets (26.5%) (*p* > 0.05).

Microscopic examination of the collected liver from Group 5 revealed maximum inhibition of inflammatory reactions and significant improvement in histological changes in the renal tissues as compared to that of Groups 2 and 3. Hepatocytes and blood sinusoids were the most closely resembled to the control group (Figure 3(L5)). There were no apparent pathological changes, and histological structures appeared closest to DEXA scans of Group 1 (Figure 3(K5)).

## 4. Discussion

The effective and rejuvenating traits of herbal therapies with shilajit have been elaborated, which is why it is referred to as a “defeater of mountains” and “destroyer of faintness”. It has been stated in the “Charaka Samhita” that many diseases, which are cured by other medical practices, can also be effectively cured by shilajit when administered at an appropriate time, either independently or in combination with other drug therapies. Studies have shown that when shilajit is administered to individuals suffering from an ailment, it helps in rejuvenating energy, while curing the disease.

The biochemical and structural analyses of the molecules used validated their biochemical characteristics and structures. From the NCT spectrum (Figure 1A), the nano-biopolymer had the main distinctive bands of the ordinary chitosan [20]. The N–H and O–H molecular hydrogen bonds are the frequent TPP locations for interactions with chitosan and synthesizing NCT [21]. The designative bands in the NCT spectrum were closely reported throughout several relevant investigations [15,22,23]. The detected peaks at 1144 and 1022 cm^−1^ correspond to the overlapping of C–O after interaction between NH_4_ groups in NCT and PO_4_ and the corresponding peak to the stretched P=O bond, confirmed NCT synthesis after interaction with TPP [15,21,22]. The detected bonds/groups in the SWE spectrum are in accordance with numerous reports [24,25,26]. The detected peaks in the SWE spectrum clearly validated the occurrence and predominance of fulvic acids in the SWE [24,27].

During the present study, GIO-afflicted rats experienced increased oxidative stress as compared to non-porotic controls, which was evident due to increased levels of MDA. This study showed changes in oxidative stress markers in treated rats, hence, it suggests shilajit treatments offering potent effects on bone regeneration by way of antioxidative and anti-osteoporotic activities. This phenomenon was notable particularly for those animals that were receiving high dose of shilajit (250 mL/kg for 8 weeks). These individuals had reduced lipid peroxidation and higher concentrations of measured antioxidant biomarkers as compared to those, which received no treatments. We found a significant decrease in TAC and SOD signaled by reduced serum measurements in GIO-afflicted rats (Groups 2, 3, and 4). Our studies on shilajit confirm earlier studies by revealing that differentiation and function of osteoclasts were stimulated by ROS, more particularly by H_2_O_2_ and superoxide anion [28,29,30,31]. During bone resorption, NADPH oxidase is expressed by osteoclasts to generate ROS in large quantities. Earlier studies showed that enzymatic antioxidants (e.g., SOD, GSH-Px, and glutathione S transferase) were decreased and lipid peroxidation and H_2_O_2_ levels increased in ovariectomized rats [32,33]. This has led to assuming that H_2_O_2_ was essential for osteoclast differentiation [34,35]. Similarly, osteoporosis in ovariectomy animals caused a significant reduction in glutathione and thioredoxin levels [28]. On the other hand, the activities of glutathione and thioredoxin reductase were also reduced in rodents [36].

Antioxidants kill free radicals without turning themselves into free extremes. Shilajit is a type of antioxidant, which is intense on the one hand and possesses significant potential on the other to cross the blood–cerebrum boundary. As reported, it also possesses the quality of free radical rummaging [34]. The antioxidant effects of shilajit depend on its concentrations; the higher doses give more prominent free radical protection [1,3,35]. The present study provides novel biochemical evidence suggesting shilajit plays a significant role in maintaining proper oxidant and antioxidant status for GIO-afflicted rats via its remarkable antioxidant properties. This is in accordance with the recent studies made on experimental models revealing beneficial traits of shilajit, which may further strengthen the key mechanism for decreasing oxidative stress (MDA and H_2_O_2_). Shilajit also stimulates osteoblastic differentiation for mesenchymal stem cells and acts as an inhibitor of osteoclastogenesis [37].

Tripathi et al. reported that ellagic acid, fulvic acid, and dibenzo-α-pyrones, the main antioxidant ingredients of shilajit, protect tissues from the damages caused by lipid peroxidation and free radicals [38]. Fulvic acid possesses scavenging properties of superoxide anion (O_2_^−^.) and hydroxyl radical and similarly, ONOO^−^ (a product of NO and O_2_^−^) suppressed differentiation of osteoblasts [39], suggesting antiradical activities of shilajit on the differentiation of osteoplastic in mesenchymal precursor cells; a fact that conforms to our present findings. Shilajit is also known to reduce dehydroascorbic acid to ascorbic acid due to the presence of dihydroxybenzo-α-pyrones [34,40]. The role of ascorbic acid is very important since it accelerates osteoblast differentiation, collagen synthesis and deposition, and bone cell mineralization [41].

During the present study, serum calcium, phosphorus, and CT levels were decreased in GIO-afflicted rats as compared to the control group. We also found a slight increase in the level of these parameters for low-dose shilajit treatment rats and a marked increase in the level of these parameters for rats receiving high-dose treatment. The above phenomenon confirms our findings, where shilajit inhibited osteoclastogenesis but stimulated osteoblastic differentiation. Similar results were also obtained by previous workers [37]. Likewise, shilajit was reported to heal fractured bones in children with a daily oral dose of 0.1 g/kg, which accelerated phosphorus uptake and callus formation in the fractured bones [5]. Various dosages of shilajit healed fractured bones differently, but the one resulting in the maximum healing in experimental rats was to be started at the earliest onset of fracture at a daily oral dose ranging between 260 and 300 mg/kg for less than seven days [2;37]. These studies suggest a positive role of shilajit, which alters calcium metabolism due to the presence of glucocorticoid by causing significant bone rejuvenation. Suppressed rates of calcium excretion and OC in GIO-afflicted rats suggest that shilajit can prevent glucocorticoid-induced bone turnover and restore calcium homeostasis.

Osteoclastic activity is a biochemical indicator used to determine the degree of bone resorption; when applied to the present study to evaluate bone resorption, OC levels were found significantly higher prior to glucocorticoid induction, which is suggestive of accelerated bone turnover. Hence, our results are in conformity with others who also reported increased levels of serum OC associated with increased bone formation [42]. Therefore, our study presents a novel finding where OC levels were significantly suppressed when shilajit was administered at higher doses.

Pro-inflammatory cytokines (such as IL-6) are known to differentiate osteoclast precursors and osteoclast activity, giving rise to bone resorption [43]. Our study showed that in GIO experimental rats IL-6 increased for enhanced bone resorption and accelerated bone turnover, which is in conformity with the earlier workers [44]. Additionally, other workers also showed increased levels of IL-6 levels under different medical conditions such as arthritis, periodontal disease, bone loss, rheumatoid arthritis, Paget’s disease, multiple myeloma, and hyperparathyroidism [45,46,47]. During the present study, IL-6 levels were most effective for the rats who received higher shilajit doses as compared to those administered with low doses. Our observations on the decrease in the production of reactive oxygen and in the amount of cytokines due to shilajit treatment suggest a positive relationship between IL-6 and oxidative stress, which is in conformity with other researchers [9,48,49]. The present study further reveals acceleration in bone regeneration, reduction in inflammation, and improvement in bone healing due to shilajit therapy.

Pathological changes were observed in the liver and kidney of the rats with osteosarcoma; these changes impaired the functions of the affected organs. Ballooning degeneration of hepatocytes with moderate inflammation and sinusoidal congestion were found in the hepatic tissues. This allowed the destruction of numerous hepatic cells resulting in a decreased hepatic capacity of synthesizing proteins, whereas the renal tissues have demonstrated shrinkage of glomerular tufts with moderate tubular atrophy. The hepatic inflammation accompanied by an increase in IL-6 levels was an inflammatory marker. Inflammatory responses were orchestrated and affected by cytokines and chemokines, which are when decreased should diminish overall inflammation [50,51]. Shilajit treatment showed overall improvement in the pathological changes observed in the liver and kidney. Inflammatory markers were also notably decreased in treated rats, which are attributed to the anti-inflammatory properties of shilajit.

Our results showed positive correlations between estimated antioxidants, SOD and TAC; and negative correlations between oxidative stress biomarkers MDA, and estimated antioxidants (SOD and TAC) (Table 3). Our findings of the above correlations are in conformity with previous studies, which also showed decreased oxidative stress and increased antioxidant values after shilajit treatment in an osteoporotic model. This phenomenon also explains why increased H_2_O_2,_ and MDA levels could not be detected following shilajit treatment with different doses. The osteoporotic women had lipid peroxidation due to increased oxidative stress and increased MDA levels, a finding that confirms our study with shilajit showing a highly negative correlation between SOD and MDA. Oxidative stress may be the cause of changes in protein and nucleic acid structures, lipid peroxidation, intracellular calcium levels, membrane permeability, etc., thus it may be the cause of several pathological conditions [52].

Glucocorticoid application rejuvenates the bone and its physiology while affecting calcium homeostasis due to a decrease in calcium and phosphorus reabsorption, which might weaken the strength in the tibia leads to osteoporosis in the rats. Pro-inflammatory cytokines (e.g., IL-6) secreted due to these pathological events, and increased levels of MDA due to lipid peroxidation, reduce antioxidant levels such as SOD and TAC. High dose shilajit treatment demonstrated an osteoprotective effect in animal models of osteoporosis, where bone integrity was primarily involved due to increased calcium, phosphorus, and CT levels. In other words, antioxidant parameters were improved due to the osteogenic effects of SWE [5]. Treatments of osteoporosis with SWE and SWE/NCT open new doors and novel perspectives to achieve positive results and benefit osteoporotic patients.

## 5. Conclusions

Shilajit is a potent and safe dietary supplement with the potential of treating and curing several diseases including osteoporosis. Shilajit and SWE loaded onto NCT in animal (rats) feed were investigated as potential cures for osteoporosis. The SWE/NCT nanocomposite was successfully generated and characterized; their nanoparticle size was approximately 248.4 nm, respectively, with high positive charging and stability. The treatments could beneficially improve osteoporosis indicators; enhance calcium, phosphorus, osteocalcin, and calcitonin levels; reduce hydrogen peroxide, and IL-6, and antioxidant biomarkers. The nanocomposite (SWE/NCT) gave the most effectual results. The formulation of SWE/NCT could be recommended as a potential innovative cure for osteoporosis. Further investigations of SWE/NCT biosafety, applicability, and action modes are suggested for this innovative nanocomposite, leading to therapeutic advances for the treatment of osteoporosis by stimulating antioxidants and decreasing oxidative stress.

## Figures and Tables

**Table 1:** Serum concentrations of calcium (ca), phosphorus (p), osteocalcin (OC), calcitonin (CT), and interleukin-6 (IL-6) in the control and treatment groups.

Groups *	Biochemical Parameters **				
	Ca mg/dL	P mmol/L	OC ng/mL	CT pg/mL	IL-6 ng/mL
Group (1)	9.81 ± 0.318 ^a^	6.00 ± 0.554 ^a^	5.30 ± 0.394 ^a^	4.96 ± 0.194 ^a^	6.41 ± 0.331 ^a^
Group (2)	7.93 ± 0.216 ^b^	3.61 ± 0.392 ^b^	9.60 ± 0.651 ^b^	2.01 ± 0.210 ^b^	21.13 ± 1.433 ^b^
Group (3)	8.10 ± 0.126 ^ab^	4.58 ± 0.213 ^ab^	7.45 ± 0.398 ^ab^	2.85 ± 0.149 ^ab^	12.28 ± 0.348 ^ab^
Group (4)	8.70 ± 0.236 ^ab^	5.30 ± 0.282 ^ab^	6.40 ± 0.328 ^ab^	3.86 ± 0.190 ^ab^	9.20 ± 0.316 ^ab^
Group (5)	9.52 ± 0.134 ^a^	5.82 ± 0.437 ^a^	5.95 ± 0.312 ^ab^	4.48 ± 0.196 ^ab^	7.92 ± 0.428 ^ab^

* Study groups (6 animals each) included the negative control (Group 1), the positive control (Group 2), shilajit treated with a dose of 150 mg/kg (Group 3), shilajit treated with a dose of 250 mg/kg (Group 4) and shilajit/nanochitosan treated with a dose of 500 mg/kg (Group 5). ** Results are expressed as the means ± SD. ^a^ Shows a statistically significant difference (*p* < 0.05) as compared to Group 2. ^b^ Shows a statistically significant difference (*p* < 0.05) as compared to Group 1.

**Table 2:** Concentrations of bone tissue oxidative stress parameters and antioxidant biomarkers in the control and treatment groups.

Groups *	Biochemical Parameters **			
	SOD (U/L)	TAC (mM/L)	MDA (nmol/mL)	H_2_O_2_ mM/L
Group (1)	307.83 ± 15.67 ^a^	2.48 ± 0.125 ^a^	2.53 ± 0.408 ^a^	1.33 ± 0.249 ^a^
Group (2)	137.33 ± 15.97 ^b^	0.7467 ± 0.092 ^b^	6.68 ± 0.318 ^b^	4.25 ± 0.278 ^b^
Group (3)	186.33 ± 6.77 ^ab^	1.10 ± 0.186 ^ab^	5.50 ± 0.334 ^ab^	3.30 ± 0.228 ^ab^
Group (4)	247.00 ± 7.69 ^ab^	2.05 ± 0.134 ^ab^	4.08 ± 0.331 ^ab^	2.44 ± 0.301 ^ab^
Group (5)	293.66 ± 12.61 ^b^	2.41 ± 0.131 ^b^	3.15 ± 0.411 ^ab^	1.53 ± 0.292 ^b^

* Study groups (6 animals each) included the negative control (Group 1), the positive control (Group 2), shilajit treated with a dose of 150 mg/kg (Group 3), shilajit treated with a dose of 250 mg/kg (Group 4) and shilajit/nanochitosan treated with a dose of 500 mg/kg (Group 5). ** Results are expressed as the means ± SD. ^a^ Shows a statistically significant difference (*p* < 0.05) as compared to Group 2. ^b^ Shows a statistically significant difference (*p* < 0.05) as compared to Group 1.

**Table 3:** Correlation coefficient (r) values for some measured parameters in all groups *.

	Ca	P	IL-6	OC	CT	MDA	H_2_O_2_	SOD	TAC
Ca		0.811	0.792	0.797	0.925	0.906	0.899	0.890	0.890
P	0.811		0.906	0.851	0.902	0.916	0.914	0.900	0.909
IL-6	0.792	0.906		0.938	0.917	0.913	0.924	0.910	0.886
OC	0.797	0.851	0.938		0.936	0.909	0.917	0.931	0.906
CT	0.925	0.902	0.917	0.936		0.966	0.964	0.970	0.954
MDA	0.906	0.916	0.913	0.909	0.966		0.951	0.959	0.947
H_2_O_2_	0.899	0.914	0.924	0.917	0.964	0.951		0.958	0.931
SOD	0.890	0.900	0.910	0.931	0.970	0.959	0.958		0.961
TAC	0.890	0.909	0.886	0.906	0.954	0.947	0.931	0.961	

* Correlation was deemed significant at the 0.01 level (2 tailed).

## Data Availability

The data presented in this study are available on request from the corresponding author.
